# Effects of NK-4 in a Transgenic Mouse Model of Alzheimer's Disease

**DOI:** 10.1371/journal.pone.0030007

**Published:** 2012-01-04

**Authors:** Hitomi Ohta, Shigeyuki Arai, Kenji Akita, Tsunetaka Ohta, Shigeharu Fukuda

**Affiliations:** Research Center, Hayashibara Biochemical Laboratories, Inc., Okayama, Japan; Case Western Reserve University, United States of America

## Abstract

Beta-amyloid (Aβ) peptides are considered to play a major role in the pathogenesis of Alzheimer's disease (AD) and molecules that can prevent pathways of Aβ toxicity may be potential therapeutic agents for treatment of AD. We have previously reported that NK-4, a cyanine photosensitizing dye, displays neurotrophic and antioxidant activities. In this study, we report the effects of NK-4 on the toxicity of Aβ and on cognitive function and Aβ concentration in a transgenic mouse model of AD (Tg2576). *In vitro*, NK-4 effectively protected neuronal cells from toxicity induced by Aβ. In addition, it displayed profound inhibitory activities on Aβ fibril formation. *In vivo*, Tg2576 mice received an intraperitoneal injection at 100 or 500 µg/kg of NK-4 once a day, five times a week for 9 months. Administration of NK-4 to the mice attenuated impairment of recognition memory, associative memory, and learning ability, as assessed by a novel object recognition test, a passive avoidance test, and a water maze test, respectively. NK-4 decreased the brain Aβ concentration while increasing the plasma amyloid level in a dose-dependent manner. NK-4 also improved memory impairments of ICR mice induced by direct intracerebroventricular administration of Aβ. These lines of evidence suggest that NK-4 may affect multiple pathways of amyloid pathogenesis and could be useful for treatment of AD.

## Introduction

Alzheimer's disease (AD) is the most common cause of dementia in elderly people and affects more than 25 million individuals worldwide. The development of therapeutic strategies for AD is a major unmet medical need. Inhibition of acetylcholinesterase (AChE) is currently a primary therapeutic strategy for the mid to late phase of AD because restitution of a close to normal acetylcholine concentration in the synaptic cleft to enhance cholinergic neurotransmission could ameliorate symptoms of AD. However, the therapeutic effect of AChE inhibitors is only symptomatic and short-term, and therefore new treatments are needed as disease-modifying therapy for AD.

Elevation of the levels of β-amyloid (Aβ), a ∼4 kDa secreted polypeptide, is thought to be involved in the pathogenesis of AD because mutations in the Aβ precursor protein (AβPP) or presenilin genes that lead to increased production of Aβ are causative for familial AD [1]-[8]. Elevated levels of Aβ are also well correlated with cognitive decline in the early phase of dementia [9]. This indicates that suppression of Aβ in the brains of patients in the early phase of dementia is a primary therapeutic target. The majority of new treatments aim to inhibit Aβ toxicity. These include Aβ immunotherapies that enhance clearance of accumulating Aβ in brain and secretase inhibitors that inhibit production of Aβ from AβPP. Inhibitors of Aβ aggregation are also currently under development, because the secondary structure determines several important properties of Aβ that may be relevant to the pathogenesis of AD.

The Tg2576 mouse model of AD expresses a high level of the Swedish mutation of AβPP under control of the hamster prion protein promoter, which leads to elevated Aβ production and accumulation in brain [10], [11]. The Tg2576 strain does not exhibit aggressive neuronal degeneration, but the mice display age-dependent cognitive dysfunction [10], [12]–[14] and are therefore an excellent tool for evaluation of Aβ-targeted pharmacological agents.

NK-4 is a divalent cationic pentamethine trinuclear cyanine dye that contains three quinolinium rings, short N-alkyl side chains (C2) and two iodine anions. NK-4 has a variety of biological activities, including antimicrobial [15], macrophage-activating [16], and anticancer properties, and is used as an immunomodulator in treatment with antiviral and anticancer agents [17]. NK-4 also has anti-inflammatory properties [18] and has been used to treat allergy [19].

Recently, we found that NK-4 was a potent neurotrophic agent for promotion of growth and differentiation of neuronal PC12 cells. The neuroprotective effects of NK-4 are mediated by activation of PI3K-Akt and inactivation of SAPK/JNK independently of the TrkA receptor at nanomolar concentrations [20]. NK-4 is also a potent scavenger of hydroxyl radicals (IC_50_: 7.6 µM), peroxy radicals (IC_50_: 5.2 µM), and superoxides (IC_50_: 89.3 µM), and these activities are greater than those of ascorbate [21]. *In vivo*, NK-4 has been shown to be effective for cerebral injury in a rat model of middle cerebral artery occlusion [21] and for motor discoordination and cerebellar atrophy in a genetic animal model of cerebellar ataxia, without causing significant adverse reactions [20].

In the present study, we investigated the effects of NK-4 on AD *in vitro* and *in vivo*. We show that NK-4 is effective against Aβ_25–35_-induced cytotoxicity in PC12 cells, and that NK-4 directly inhibits fibril formation of three forms of Aβ peptide (Aβ_25–35_, Aβ_1–40_ and Aβ_1–42_). In AβPP Tg mice, NK-4 significantly improved cognitive dysfunction in association with a substantial decrease in the Aβ-immunoreactive tangles and Aβ concentrations in the brain. Moreover NK-4 reversed cognitive impairments induced by an intracerebroventricular injection of Aβ_25–35_ in mice.

## Methods

### Chemicals

NK-4 (4,4′-[3-{2-(1-ethyl-4(1H)-quinolylidene)ethylidene}propenylene] bis(1- ethylquinolinium iodide)) was synthesized at Hayashibara Biochemical Laboratories, Inc. (Okayama, Japan). A stock solution of 5 mg/ml NK-4 was prepared in DMSO and stored at room temperature with protection from light. Just before use, the stock solution was diluted with medium to give a 25 µg/ml solution. This working solution was used for experiments with further dilution. Aβ_25–35_, Aβ_1–42_ and Aβ_1–40_ were obtained from Anaspec (San Jose, CA). All other chemicals were purchased from Sigma-Aldrich (St. Louis, MO), unless otherwise indicated.

### Cell Culture

The rat pheochromocytoma cell line PC12 (Human Science Research Resources Bank, Osaka, Japan) was cultured in Dulbecco's Modified Eagle Medium (D-MEM; Nissui, Tokyo, Japan) supplemented with heat-inactivated 10% (v/v) fetal bovine serum and 5% (v/v) horse serum. For cell growth and neurite-outgrowth assays, PC12 cells were harvested using 0.1% (w/v) trypsin containing 0.03% (w/v) EDTA and seeded at a density of 5,000 cells/100 µl in 96-well plates pre-coated with collagen type IV. After a 24 hr pre-culture, PC12 cells were exposed to Aβ_25–35_ (50 µM) in the presence or absence of NK-4 for 72 hr. Aβ_25–35_ peptide were dissolved in DMSO (2 mM) and kept at −80°C. Just before use, the solution was diluted in distilled water (200 µM) and incubated at 37°C for 16 hr. This procedure, called “aging”, promotes formation of stable oligomeric aggregates and enhances the cytotoxicity of the preparation. Cell survival was determined using alamarBlue dye (Trek Diagnostic Systems, Cleveland, OH) [22].

### Thioflavin T (ThT) Fibril Formation Assay

The Aβ fibril formation assay was performed as described previously [23]. Stock solutions of Aβ_1–40_, Aβ_1–42_ and Aβ_25–35_ were prepared in DMSO (1 mg/ml) and stored at −80°C. Just before the experiment, the stock solution was diluted with 10 mM phosphate buffer containing 100 mM KCl (pH 7.4). The diluted Aβ solutions were incubated at 37°C in the presence or absence of indicated concentrations of NK-4 for 72 hr. Fifty µl of the incubated samples containing 100 µM Aβ peptides were added to 450 µl of 10 µM ThT in 10 mM phosphate and 100 mM KCl (pH 7.4). Fluorescence intensity was measured with excitation at 435 nm and emission at 485 nm using a Hitachi 650-60 fluorescence spectrophotometer. The results are expressed as the mean ± SD of three samples.

### Electron Microscopy

Amyloid fibril formation was verified by electron microscopy (EM) images of negatively stained samples. A 20-μl sample of Aβ_1–40_ or Aβ_1–42_ solution incubated with or without NK-4 was placed on a polyvinyl butyral-coated copper mesh grid (Oukenshoji, Tokyo, Japan). The sample was allowed to stand for 30–60 s and excess solution was washed away. Subsequently, samples were negatively stained with 2% (w/v) uranyl acetate (Kowayakuhin, Saitama, Japan) and allowed to dry. After staining, the samples were viewed with a JEM-100CX/II electron microscope (Joel Ltd., Tokyo, Japan) at 80 kV.

### Animals

All animal protocols were approved by the Institutional Animal Care and Use Committee of Hayashibara Biochemical Laboratories (permit number F-1002) and were conducted in accordance with the guidelines for the Care and Use of Laboratory Animals at Hayashibara Biochemical Laboratories. Female AβPP transgenic mice (Tg2576, 129S6/SvEvTac background) expressing a double mutation of human AβPP [24], and age- and sex- matched non-transgenic mice were obtained from Taconic (Germantown, NY). The mice were housed individually with supplied nesting materials, and food and water were provided ad libitum. NK-4 solution was injected intraperitoneally at a dose of 100 or 500 µg/kg once a day, five times a week for 9 months, beginning at 3 months of age. Control mice received 200 µl of saline. During the behavioral test periods, NK-4 or saline was administered 2∼3 hr before the start of each test. Male ICR mice (5 weeks of age, weighing 25–30 g) were purchased from Charles River Japan (Kanagawa, Japan).

### Aβ25-35-Induced Cognitive Impairments in ICR Mice

Cognitive impairment was induced by a direct intoracerebroventricular (icv) administration of aggregated Aβ_25–35_ peptide in mice according to the method described by Maurice et al. [25]. Aβ_25–35_ was dissolved in saline at the concentration of 1.0 mM and incubated at 37 °C for 4 days, called “aging” process. On day0, aged Aβ_25–35_ solution (9 nmole/6 µl/mouse) was injected by a micro-stainless needle (inner diameter: 0.13 mm) into the left lateral ventricle of ICR mice using following coordinates from Bregma: 0.5 mm posterior, 1.0 mm lateral, and 2.0 mm ventral. Sham operated mice received 6 µl of saline instead of Aβ_25–35_ solution. From the next day of Aβ-icv injection (day1), NK-4 solution was injected intraperitoneally at a dose of 50 or 500 µg/kg/day for twelve consecutive days. Control mice received 200 µl of saline. Mice were tested for object recognition and passive avoidance according to the methods described below during the day6–8 and day9–12, respectively.

### Novel Object Recognition Test

The task was carried out according to a previous report [26] with minor modification. The apparatus used in this study was an opaque plastic box (40×50×30 cm) with a clear acrylic cover plate on the top, the floor of which was covered with wood tips. The novel object recognition task comprised the following three sessions. First, in a habituation session, mice were individually placed in the box for 10 min of exploration in the absence of objects. Second, a training session was performed 24 hr after the habituation session. Two objects (A and B) were placed in the back corner of the box, 10 cm from the sidewall. Mice were individually placed in the middle front of the box and the time spent in exploring the two objects was recorded. Exploration behavior of the mice was defined as directing the nose toward the object at a distance of less than 2 cm. Third, a retention session was performed 24 hr after the training session. One of the familiar objects (B) used in the training session was replaced by a novel object C, and objects A and C were placed as in the previous session. Mice were then individually placed in the box and allowed to explore freely for 10 min, with recording of the time spent in exploring each of the two objects. The exploratory preference was expressed as a ratio of the time spent exploring the novel object (TC) over that spent on the two objects (TA+TC): exploratory preference (%)  =  TC/(TA+TC) ×100.

### Water Maze Test

A circular pool (diameter  = 130 cm, Nishinihonkikou, Okayama, Japan) was filled with water to a height of 15 cm. White ink was used to render the water opaque. The water temperature was maintained at 23±1°C throughout the trials. To enhance the positional recognition of mice, four different geometric patterns were placed at inner rim of each quadrant of the pool as well as each wall of the room. On the first day (day0), the platform (diameter  = 9 cm) was placed at the midpoint of the pool, 0.5 cm above the water surface. Each mouse was placed on the platform and allowed to move away from the platform and swim in the pool for 15 sec, then gently guided to the original platform. The next day (day1), the platform was placed at the midpoint of one quadrant, submerged 0.5 cm below the water surface. The position of the platform was unchanged throughout the test trials. Two trials each day with an inter-trial interval of 1 min were conducted for 4 consecutive days (day1 to day4). In each trial, the mice were placed in the pool at one starting position, with their back turned to the midpoint of the pool. They were allowed to swim freely or until they found the hidden platform and came up for air while on the platform. The time required to escape onto the hidden platform was recorded as escape latency. If a mouse did not reach the platform within 120 sec, it was gently guided to the platform, where it remained for 10 sec. Starting locations were changed every trial. The results of 2 trials were averaged and used in data analysis.

### Passive Avoidance Test

A passive avoidance test was conducted using the apparatus consisted of two separate chambers (30×30×20 cm height). One of the chambers was illuminated and the other was dark. Each chamber was separated by a small guillotine door (3.5×5 cm) and grids were attached on the floor in the dark chamber. This test comprised the following 3 sessions. First, in a pre-training session (day1 and day2), mice were individually placed in the illuminated chamber for 1 min and then in the dark chamber for 2 min. Second, in the test session (day3), mice were individually placed in the illuminated chamber. Immediately after the mice entered the dark chamber, the door was locked and an electrical stimulation of 0.36 mA was applied for 2 sec. The time spent in the illuminated chamber was measured. Third, in the retention session (day4), which was performed 24 hr later, mice were placed back in the illuminated chamber and the time spent in this chamber before entering the dark chamber was measured. The latency in the retention session was expressed graphically and used in data analysis.

### Measurement of Aβ Species from Plasma and Brain Homogenates by ELISA

After mice were deeply anesthetized with pentobarbital (50 mg/kg), blood samples were withdrawn from the postcaval vein using heparinized syringe and the plasma was prepared and used fresh or was stored at −80°C until use. Cerebrospinal fluid (CSF) was collected from the dura mater over the cistern magna and brains were isolated. Hemi-brains were weighed and homogenized with a teflon-glass homogenizer in 20 mM Tris-buffered saline (TBS; 137 mM NaCl, protease inhibitor cocktail, pH7.6), and centrifuged at 100,000×g for 1 hr at 4°C. Pellets were resuspended in TBS containing 2% SDS. After centrifugation at 100,000×g for 1 hr at 4°C, the supernatant was obtained as the soluble fraction of Aβ, and the pellet was extracted with 70% (w/v) formic acid, followed by centrifugation at 100,000×g for 1 hr at 4°C. The supernatant was neutralized by 20-fold dilution with 1 M Tris base [27] and used as the insoluble Aβ fraction. Samples were diluted with the diluents provided in the human (h)Aβ_1–40_ and hAβ_1–42_ ELISA kits (Wako, Osaka, Japan). hAβ_1–40_ and hAβ_1–42_ in individual samples were quantified in accordance with the manufacturer's instructions and are expressed as picomoles/mL of plasma or picomoles/mg wet weight of brain (mean ± SEM).

### Histology

Hemi-brains were fixed with 10% buffered formalin for 24 hr and embedded in paraffin blocks, and then coronal sections of cerebral cortex with 5 µm thickness were stained with Congo-Red for counting amyloid plaques. For Aβ immunohistochemistry, paraffin sections were dewaxed, rehydrated, and then blocked with PBS containing 0.2% Triton X-100 and 5% BSA. Sections were then incubated with rabbit anti-Aβ-peptide polyclonal antibody (#71-5800, 1∶50) (Invitrogen, Camarillo, CA) at 4°C overnight. After several washes in PBS, sections were incubated with HRPO-conjugated secondary antibody (DAKO, anti-rabbit Igs, 1∶2000) and then Aβ-immunoreactive tangles were visualized by diaminobenzidine.

### Statistical Analysis

All values are expressed as means ± SD or SEM. A Student t test was used for comparison between 2 groups. One-way ANOVA with a Tukey-Kramer test was used to determine the significance of differences in multiple comparisons. In water maze, repeated measures two-way ANOVA followed by a post hoc Sheffe's F test was applied. Pearson correlation analysis was used to examine the correlation between Aβ levels in plasma and brain in each mouse. Differences with a probability value of P<0.05 were considered to be significant.

## Results

### Effects of NK-4 against Aβ-Induced Neurotoxicity in PC12 Cells

**Figure 1:**
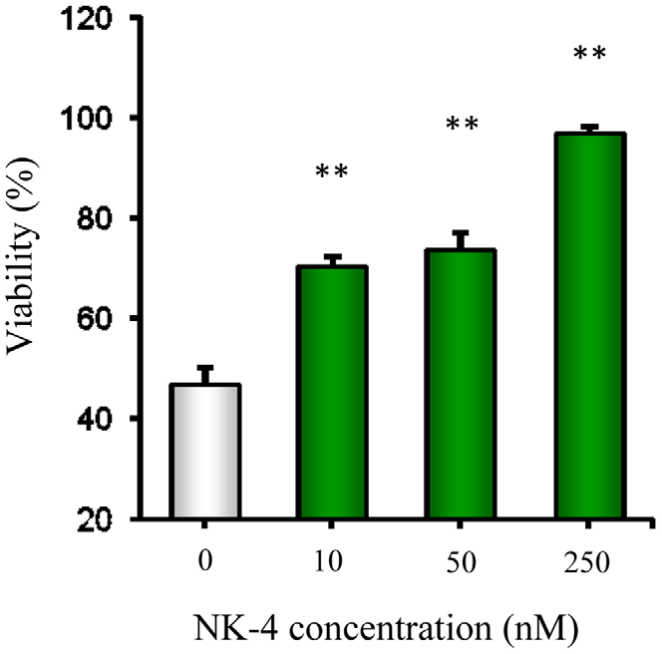
Cytoprotective effects of NK-4 on Aβ_25–35_-induced cytotoxicity in PC12 cells. PC12 cells were treated with 50 µM Aβ_25–35_ for 72 hr in the absence (open bar) or presence of the indicated concentrations of NK-4 (closed bars). Control cells were incubated under the same conditions, but without Aβ_25–35_. Cell viability was assessed by alamarBlue assay. Results are shown as means ± SD (n = 3). **P<0.01 vs. no NK-4.

We first determined whether NK-4 was effective against Aβ-induced neurotoxicity *in vitro* using PC12 cells. These cells (either undifferentiated or differentiated with nerve growth factor) are reported to be the most sensitive to Aβ protein or the Aβ_25–35_ fragment [28]. The cytotoxicity of Aβ_25–35_ peptide is stronger than that of Aβ1–40 in both PC12 cells and hippocampal neurons, which defined residues 25–35 as the active region of the Aβ protein [28]. The cytotoxicity of Aβ_25–35_ developed rapidly and was detected within 2 hr after application, and then reached a plateau after 72 hr of incubation (data not shown). The viability of PC12 cells treated with 50 µM Aβ_25–35_ for 72 hr was about 45% compared with controls without Aβ_25–35_ (Fig. 1). NK-4 dose-dependently attenuated the effect of Aβ_25–35_ and the results were significant at doses over 10 nM. NK-4 substantially reversed the cellular damage at 250 nM.

### Effect of NK-4 on Aβ Protein Fibrillization

**Figure 2:**
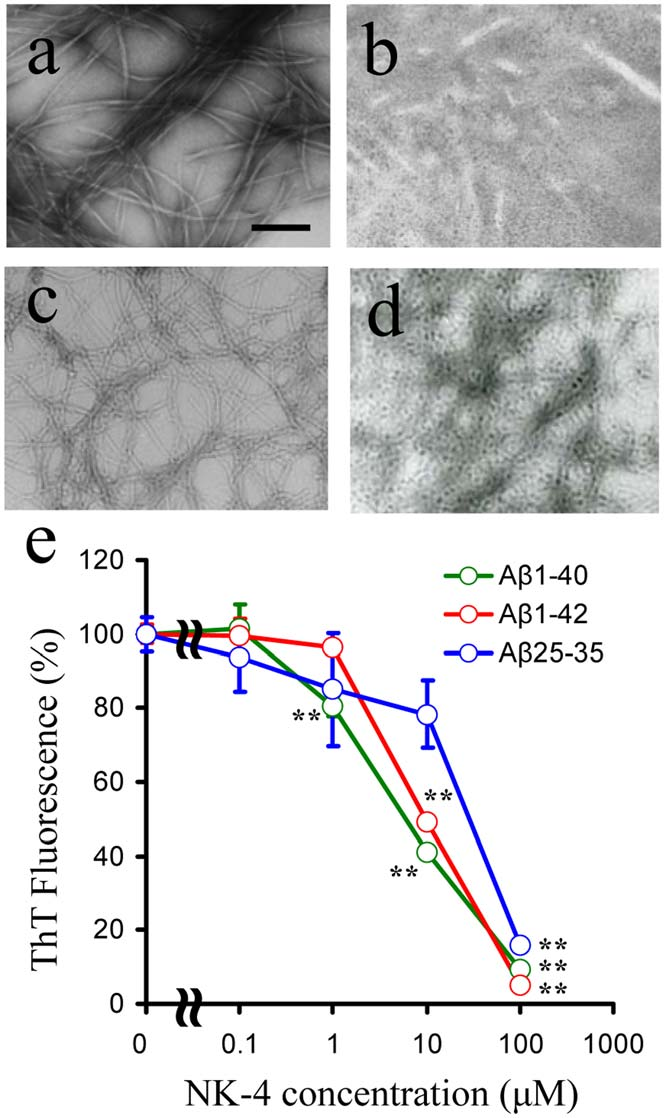
Effects of NK-4 on Aβ fibril formation. 100 µM of Aβ_1–40_ (a, b) or Aβ_1–42_ (c, d) was incubated for 72 hr alone (a, c) or with NK-4 (10 µM) (b, d). Bar represents 200 nm. The experiment was performed three times with similar results. (e) Thioflavin T assay of Aβ fibril formation. Three forms of Aβ peptide (Aβ_1–40_, Aβ_1–42_ and Aβ_25–35_) were incubated for 72 hr with the indicated concentration of NK-4. Values are means ± SD (n = 3). **P<0.01 vs. control (without NK-4).

To examine the potential mechanisms of NK-4-mediated protection of neuronal cells from amyloid toxicity, we evaluated the effect of NK-4 on aggregation or fibrillization of three forms of Aβ peptide. Solution of Aβ_1–40_ or Aβ_1–42_ (100 µM each) was incubated at 37°C for 3 days in the absence or presence of NK-4 at the indicated concentrations (Fig. 2). After the incubation, visible sedimentary aggregates were observed in the Aβ controls, while the addition of NK-4 had reduced visible aggregates. To examine the effect of NK-4 further, EM was used to monitor Aβ fibril formation. In EM, Aβ_1–40_ alone formed long and unbranched fibrils (Fig. 2a). In contrast, Aβ_1–40_ incubated with NK-4 (10 µM) had shorter and fewer filaments with soluble assemblies (Fig. 2b). Similarly, Aβ_1–42_ formed dense fibrils (Fig. 2c) and they were remarkably weakened by the co-incubation with NK-4 (Fig. 2d). ThT fluorescence is enhanced upon binding to Aβ fibrils, proportionally to the amount of fibrils in solution [23]. So, we used ThT to evaluate the effect of NK-4 on the Aβ fibril formation quantitatively. All three forms of Aβ solutions showed enhanced emission at 482 nm, which is characteristic for ThT bound to amyloid fibrils. NK-4 inhibited all these ThT fluorescence dose-dependently (Fig. 2e). The inhibitory effect of NK-4 was most prominent on Aβ_1–40_, then followed by Aβ_1–42_ and Aβ_25–35_. An equimolar concentration of NK-4 almost totally inhibited the ThT fluorescence in all three forms of Aβ. These results suggest that NK-4 is an effective inhibitor of Aβ fibril formation *in vitro*.

### Effects of NK-4 on Recognition Memory in the Novel Object Recognition Test

**Figure 3:**
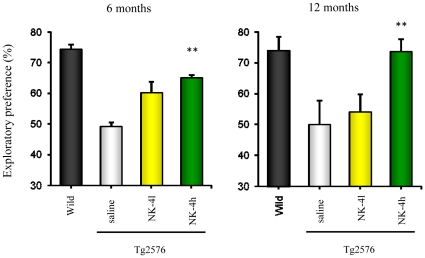
Effects of NK-4 on cognitive function of Tg2576 mice in a novel object recognition test. A low (100 µg/kg) or high (500 µg/kg) dose of NK-4 was administered intraperitoneally to female Tg2576 mice from 3 to 12 months of age. Mice were tested for object recognition at 6 and 12 months of age. Values are means ± SEM (n = 10). **P<0.01 vs. saline-treated Tg2576 group. Wild: non-transgenic female 129S6 mice. Tg2576 (saline): saline-treated Tg2576 mice. Tg2576 (NK-4l): low-dose NK-4-treated Tg2576 mice. Tg2576 (NK-4h); high-dose NK-4-treated Tg2576 mice.

Given the beneficial effects of NK-4 on multiple aspects of amyloid pathology *in vitro*, we next examined whether NK-4 had an effect on the cognitive deficit induced by Aβ in an AβPP transgenic mouse model of AD. Tg2576 mice expressing the Swedish mutation of AβPP were administered with 100 or 500 µg/kg NK-4 once a day, 5 times a week for 9 months, beginning at 3 months of age. Wild type and Tg2576 mice were tested for object recognition memory 24 hr after training to test long-term memory at ages 3, 6 and 12 months. During the training session, there were no significant differences in exploratory preference between the two objects or in the total exploratory time among the groups (data not shown). At 3 months of age, we could not detect significant differences in recognition memory among all groups tested (data not shown). In the retention session, Tg2576 mice at ages 6 and 12 months showed significantly decreased object recognition memory compared with that in the wild type mice at the respective ages (Fig. 3). Tg2576 mice treated with a low dose of NK-4 (100 µg/kg) tended to spend a longer time exploring the novel object than did the saline-treated controls at both 6 and 12 months of age (treated for 3 and 9 months), but the differences were not significant. In contrast, Tg2576 mice treated with a high dose of NK-4 (500 µg/kg) spent a significantly longer time exploring the novel object than did the saline-treated mice at both 6 and 12 months of age, and the exploratory preference was comparable to that of wild type controls at age 12 months (after 9 months treatment). This suggests that NK-4 administration for a longer period might be more effective for improvement of object recognition memory.

### Effects of NK-4 on Learning Ability in the Water Maze Test

**Figure 4:**
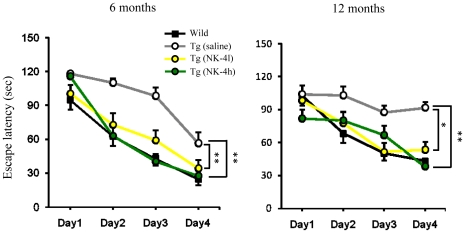
Effect of NK-4 on learning ability of Tg2576 mice in a water maze test. Mice were tested for learning ability at 6 and 12 months of age. The mean latency represents the time spent finding a hidden platform placed in a fixed location in the pool. Two trials per day were conducted for 4 consecutive days. Data are expressed as means ± SEM of the averaged time on each day (n = 10). *P<0.05, **P<0.01 vs. saline-treated Tg2576 group.

We also evaluated learning ability of mice in a water maze test at ages 6 and 12 months (Fig. 4). Group differences in the escape latency of the training trial session were analyzed using two-way ANOVA with repeated measures. There were significant differences in escape latency between genotype (wild type controls vs. saline-treated Tg2576 mice) at 6 months of age [Genotype × Time *F*(1,54)  = 40.531, P<0.001], and at 12 months of age [Genotype × Time *F*(1,48)  = 7.269, P<0.05]. For saline-treated Tg2576 group, the latency to reach the platform did not shorten during the whole test period at 12 months of age. There was a significant Genotype × Time interaction [*F*(3,48)  = 5.0732, P<0.01] at this stage, suggesting that the learning ability of saline-treated Tg2576 mice became impaired with age.

**Figure 5:**
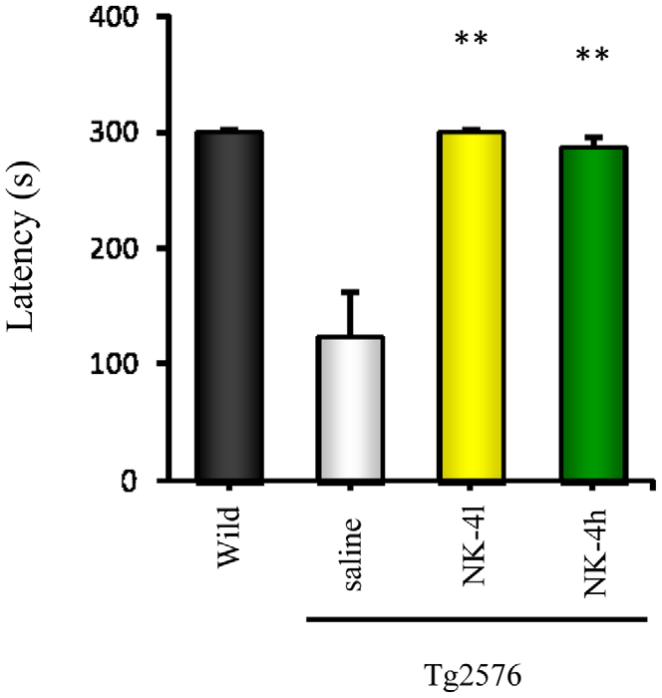
Effect of NK-4 on cognitive function of Tg2576 mice in a passive avoidance test. Mice were tested for learning at 9 months of age. The latency in the retention session performed 24 hr after the training session is shown. Values are means ± SEM (n = 10). **P<0.01 vs. saline-treated Tg2576 group.

We found significant differences in escape latency between treatments (NK-4 vs. saline) at 6 months of age [Group × Time *F*(2,81)  = 10.404, P<0.001], and at 12 months of age [Group × Time *F*(2,75)  = 5.671, P<0.01]. The interaction of the grouping factor and the time factor also proved to be significant at 6 months of age [*F*(6,81)  = 2.672, P<0.05]. Saline-treated Tg2576 group consistently exhibited a longer latency than NK-4-treated Tg2576 groups. Both low (100 µg/kg) and high (500 µg/kg) doses of NK-4 improved learning ability of Tg2576 mice with significant differences compared with saline-treated controls (P<0.01 at 6 months of age, P<0.05 at 12 months of age), but the effects of NK-4 on escape latency did not differ significantly between these two doses. The swimming ability did not differ significantly among Tg2576 groups (data not shown).

### Effects of NK-4 on Passive Avoidance Memory

Recognition memory of NK-4-treated mice at ages 3 and 9 months was evaluated in a passive avoidance test. Similar to the novel object recognition test, no significant difference was detected in latencies for entering the dark chamber of a passive avoidance apparatus during the retention session among all groups tested at 3 months of age (data not shown). However at 9 months, there was a significant difference in latencies between wild type control and saline-treated Tg2576 mice (p<0.01). Both low (100 µg/kg) and high (500 µg/kg) doses of NK-4 reversed the decrease in passive avoidance latency of Tg2576 mice, and the differences were significant compared with saline-treated controls (both p<0.01, Fig. 5). There was no difference in latencies for entering the dark chamber between wild type and Tg2576 mice, or among the treatment groups after placement in the light chamber for the first time during the training session (in the absence of shock). All animals took an average of 70 s to move to explore the dark chamber, indicating no baseline differences in anxiety in the treatment groups in this behavioral test. We also found no difference in shock sensitivity among wild type and saline- and NK-4-treated Tg2576 mice.

### Effect of NK-4 on Aβ Levels in Tg 2576 Mice

**Figure 6:**
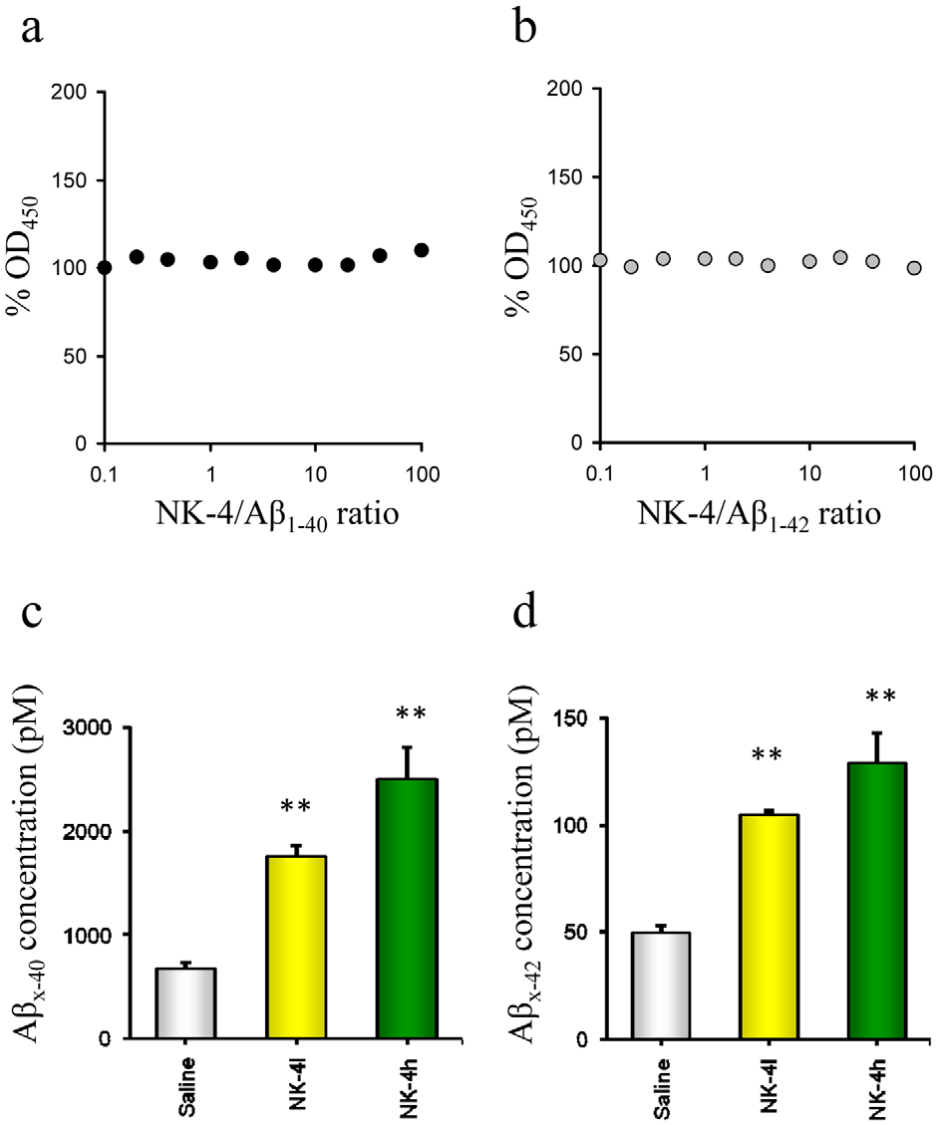
Effect of NK-4 on the plasma Aβ concentrations in Tg2576 mice. NK-4 did not interfere the optical density of both hAβ_1–40_ (a) and hAβ_1–42_ (b) ELISA systems, which using the antibody combinations of BAN50/BA27 and BAN50/BC05, respectively. Since these ELISA systems cannot distinguish N-terminal heterogeneity of Aβ peptides derived from biological samples, we represented hAβ species as hAβ_x-40_ and hAβ_x-42_, respectively. Plasma from mice aged 12 months was used for measurements of hAβ_x-40_ (c) and hAβ_x-42_ (d). Values are the mean ± SEM for saline-treated Tg2576 mice (control group, n = 10), a low dose NK-4 (100 µg/kg)-treated group (n = 9), and a high dose NK-4 (500 µg/kg)-treated group (n = 8). **P<0.01 vs. saline-treated Tg2576 group.

We next evaluated whether NK-4 displayed any significant effect on Aβ levels in plasma, CSF and brains from Tg2576 mice. Since NK-4 might directly interact with Aβ peptides, we examined whether it interferes the Aβ ELISA. Although the presence of NK-4 slightly enhanced optical density values in Aβ ELISA systems, the range of increase was less than 5% when the NK-4:Aβ molar ratio was 0.1∼100 (Fig. 6a, b). This indicated that NK-4 hardly affects both Aβ_1–40_ and Aβ_1–42_ ELISA systems.

**Figure 7:**
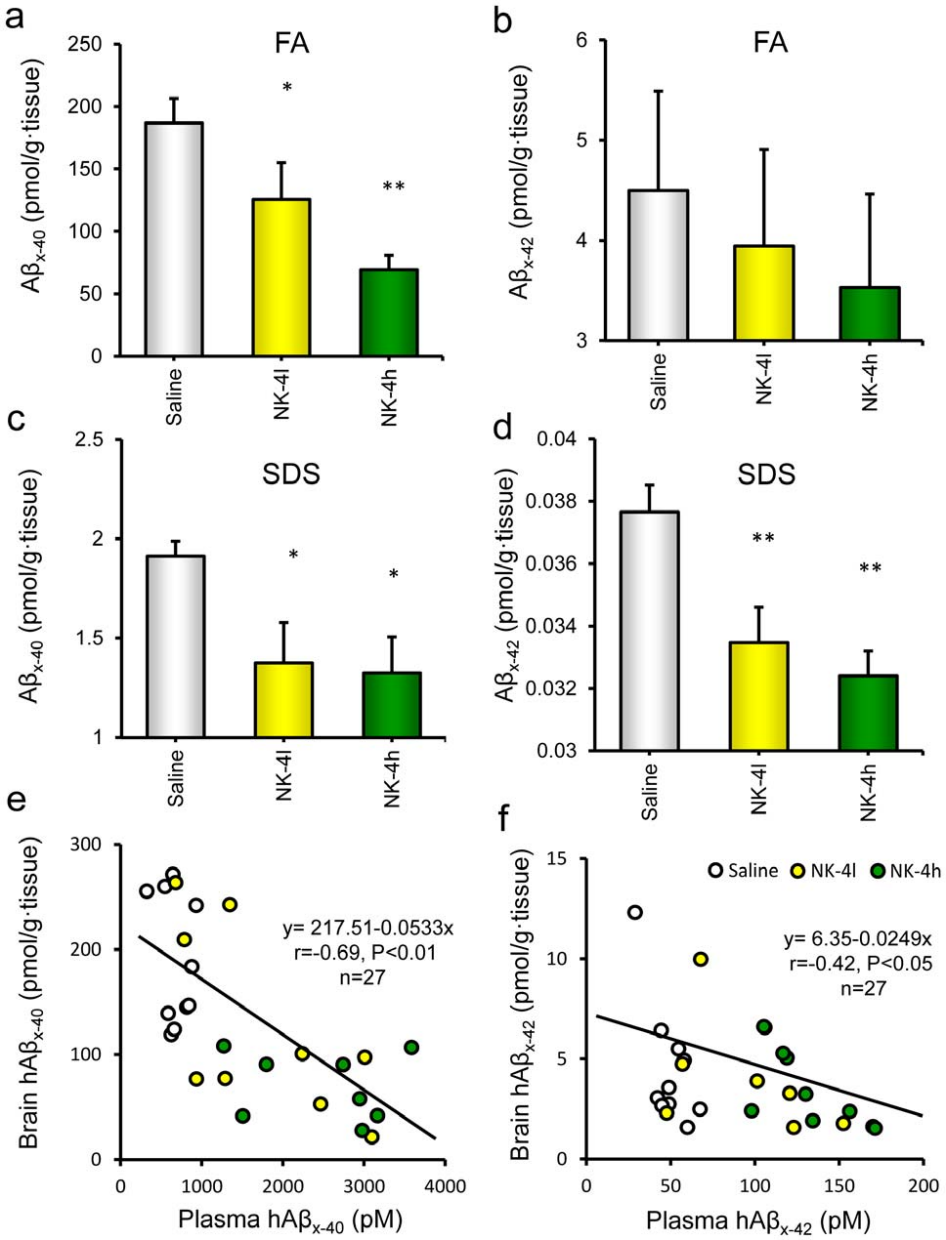
Effect of NK-4 on brain soluble or insoluble Aβ_x-40_ and Aβ_x-42_ in Tg2576 mice. Brains from mice aged 12 months were homogenized and separated into soluble (c, d) and insoluble (a, b) fractions of Aβ, and assayed for hAβ_x-40_ (a, c) and hAβ_x-42_ (b, d), respectively. FA: SDS insoluble and formic acid soluble fractions of Aβ. SDS: SDS soluble fractions of Aβ. Correlations are shown between the plasma and brain levels of hAβ_x-40_ (e) and hAβ_x-42_ (f) in Tg2576 mice. Data are the mean ± SEM of hAβ_x-40_ or hAβ_x-42_ in each mouse in saline-treated Tg2576 controls (n = 10), a low dose NK-4 (100 µg/kg)-treated group (n = 9), and a high dose NK-4 (500 µg/kg)-treated group (n = 8). *P<0.05, **P<0.01 vs. saline-treated Tg2576 group.

Dose-dependent increases in Aβ_x-40_ and Aβ_x-42_ were observed in plasma from NK-4 treated mice (Fig. 6c, d). In contrast, CSF Aβ_x-40_ and Aβ_x-42_ levels were not significantly changed by NK-4 treatment, although the CSF concentrations of Aβ_x-40_ and Aβ_x-42_ in NK-4 treated mice tended to be lower than those in saline-treated controls (data not shown). Brain levels of Aβ were estimated separately in soluble and insoluble fractions. Both soluble and insoluble Aβ_x-40_ levels were significantly decreased by NK-4 treatment (Fig. 7a, c). Similarly, levels of Aβ_x-42_ were lower in NK-4 treated mice, with a significant decrease in insoluble Aβx-42 (Fig. 7b, d). The levels of Aβ_x-40_ and Aβ_x-42_ in brain were inversely correlated with those in plasma (*r* = −0.69, P<0.01 (n = 27), and *r* = −0.42, P<0.05 (n = 27), respectively) (Fig. 7e, f).

**Figure 8:**
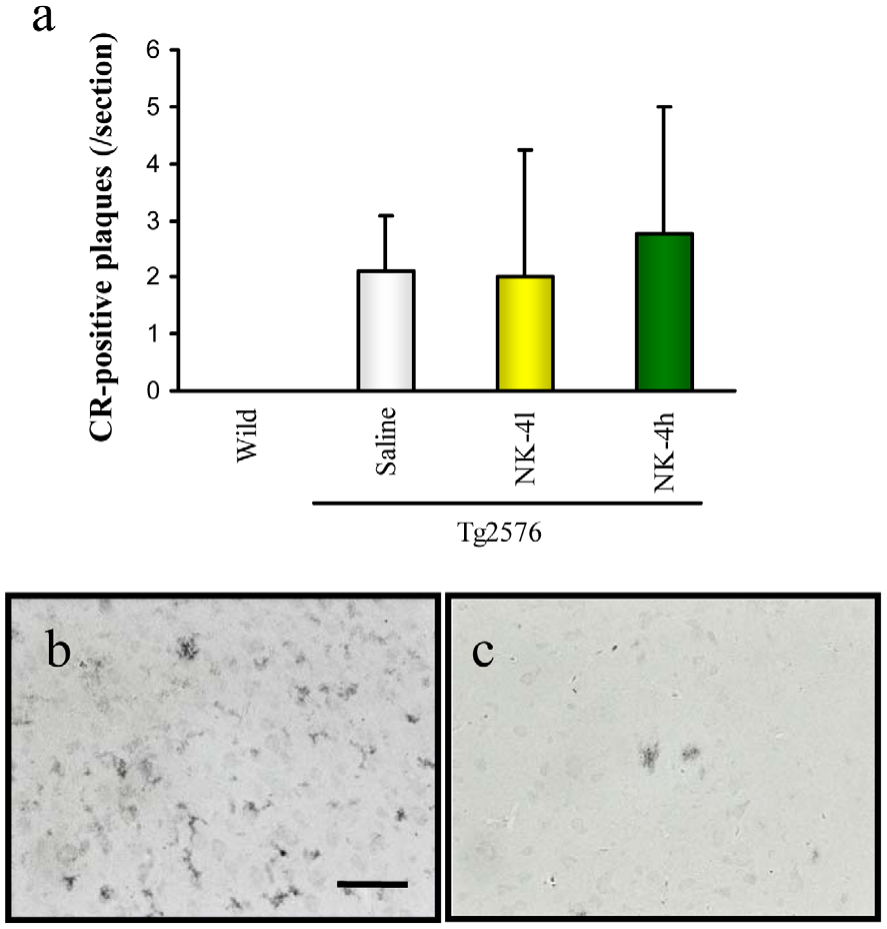
Aβ deposition in brains of Tg2576 mice at 12 months of age. Coronal brain sections (cortex region) were stained by Congo Red (a) or anti-Aβ polyclonal antibody (b, c). The number of CR-positive plaques on the section was counted in cortex region (a). Typical images of Aβ-immunoreactive tangles of saline-treated Tg2576 mouse (b) and high-dose NK-4(500 µg/kg)-treated Tg2576 mouse (c). Bar represents 50 µm.

We next surveyed brain Aβ deposition of Tg2576 mice by Congo Red (CR) staining and Aβ immunohistochemistry (Fig. 8). Wild type mice did not show any sign of amyloid deposition in their brain at 12 month of age (Fig. 8a). Tg2576 mice at the same age developed distinguishable, but very few amount of plaques in their cortex region. The number of CR-positive plaques in the cerebral cortex region was not significantly changed among saline-treated group and NK-4-treated groups (Fig. 8a). On the contrary, Aβ-immunoreactive small tangles were abundantly seen in the cortex of saline-treated Tg2576 mice (Fig. 8b), and they were obviously reduced by the high dose NK-4 treatment both in size and in quantity (Fig. 8c).

### Effects of NK-4 on Aβ25-35-Induced Cognitive Impairment in ICR Mice

**Figure 9:**
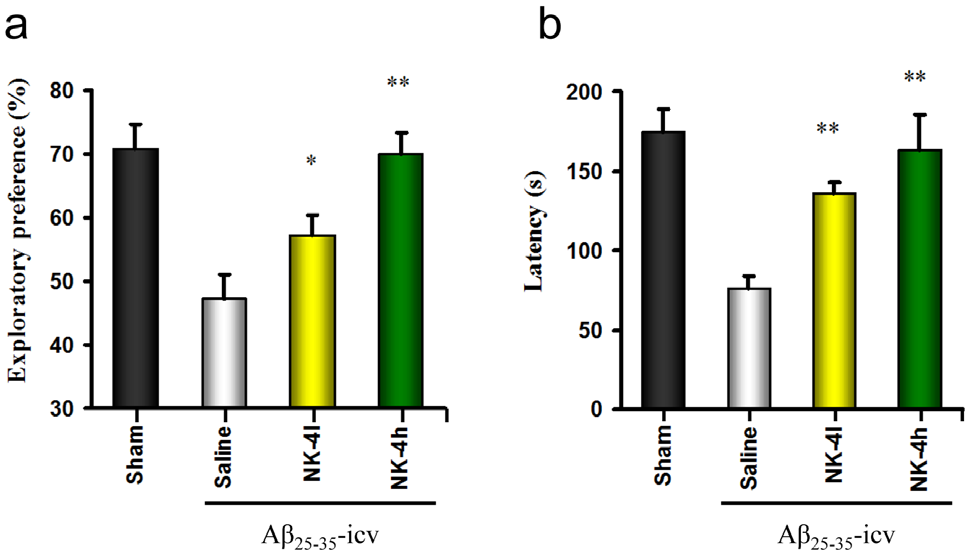
Effects of NK-4 on Aβ_25–35_-induced cognitive impairments in ICR Mice. Cognitive impairment was induced in ICR mice by icv injection of Aβ_25–35_ solution as described in Material and Method. A low (50 µg/kg) or high (500 µg/kg) dose of NK-4 was administered intraperitoneally to mice twelve consecutive days starting from the next day of Aβ injection (day1). Mice were tested for object recognition (a), followed by passive avoidance (b). Values are means ± SEM (n = 10). Sham: sham-operated group, saline: saline-treated Aβ_25–35_–icv group, NK-4l: low-dose NK-4-treated Aβ_25–35_–icv group, NK-4h; high-dose NK-4-treated Aβ_25–35_–icv group. *P<0.05, **P<0.01 vs. saline-treated group.

To confirm whether the effect of NK-4 was directly attributable to mitigation of Aβ pathology, we employed an Aβ-induced amnesia model, in which ICR mice received intracerebrovantricular (icv) administration of aged Aβ_25–35_ peptide to provoke memory deficits [25]. Long-term recognition memory was evaluated by the novel object recognition test and the passive avoidance test starting at 6 days and 9 days after Aβ_25–35_-icv injection, respectively. In both behavioral assays, daily NK-4-treatment dose-dependently, and significantly improved the memory deficits induced by Aβ (Fig. 9a, b). These results, in combination with the results from Tg2576 mice, strongly suggested that NK-4 treatment improved Aβ-mediated memory impairments.

## Discussion

NK-4 is a N-heterocyclic cationic dye derivative that contains three quinolinium rings and two iodine anions. Although NK-4 shows a variety of biochemical and biological activities [15]–[21], structure-activity relationships or molecular mechanisms of them remain largely unknown. Extended π-electron conjugated system of NK-4 would considerably contribute to antioxidative effects by trapping free radicals similar to another cyanine dye platonin [29]. The extended conjugated system also confers this molecule a planar structure by which NK-4 seems to bind at the interface of the β-sheet domains of the proteins and act as a “β-sheet breaker” [30]. On the other hand, it is reported that the side chain length affects water solubility or affinity to the lipid membrane in this kind of dye compounds [31]; therefore, the specific activity of NK-4 might change in accordance with the length of N-alkyl side chains.

Application of neurotrophic small molecules that modulate neuronal survival and synaptic function is a promising therapeutic approach for AD, while modulation of the amyloid pathology is a key strategy for slowing or halting the disease progression. In this study, we showed that NK-4 protected neuronal cells from Aβ_25–35_-induced toxicity (Fig. 1). Attenuation of Aβ cytotoxicity by NK-4 might be caused by direct action on Aβ to prevent its aggregation into more toxic forms, or via activation of intracellular survival signaling pathways and inactivation of death pathways in neuronal cells [20], or both. The effect of NK-4 on Aβ fibril formation was significant, but the IC_50_ value for Aβ aggregation was approximately 10 µM for Aβ_1–40_ and Aβ_1–42_, and 30 µM for Aβ_25–35_ (Fig. 2e). In contrast, a much lower concentration (nM level) of NK-4 attenuated the neurotoxicity induced by 50 µM Aβ_25–35_ in PC12 cells (Fig. 1). These results suggest that NK-4 attenuates Aβ-induced toxicity via activation of intracellular survival signaling pathways including Akt activation [20], rather than direct inhibition of Aβ aggregation, since the concentration of NK-4 required inhibiting Aβ aggregation was substantially high. However, the effect of NK-4 on Aβ aggregation was greater than those of non steroidal anti-inflammatory drugs (NSAIDs) such as ibuprofen, naproxen, ketoprofen, and indomethacin [32], [33], which require at least a threefold higher concentration to inhibit Aβ aggregation. Therefore, the anti-fibrillization effect of NK-4 might play some role in attenuating Aβ toxicity *in vivo*.

Chronic administration of NK-4 to Tg2576 mice significantly attenuated cognitive decline as assessed in a set of behavioral tests (Fig. 3,4,5), and also decreased the levels of Aβ in brain (Fig. 7) and augmented those in plasma (Fig. 6). These results imply that the NK-4-induced behavioral improvement was attributable to decreased Aβ in brain, and that Aβ may be cleared across the blood-brain barrier to the blood. A similar large increase in plasma Aβ was found after peripheral administration of an anti-Aβ antibody in an AD mouse model [34]. Although there is still no consensus as to the mechanism by which anti-Aβ antibodies alter amyloid deposition, *in vivo* binding properties of the antibodies would affect plasma Aβ levels by altering the half-life of Aβ [34]. In a similar fashion, a direct NK-4 binding to Aβ in Tg2576 mice may be one possible explanation of augmented plasma Aβ levels by chronic peripheral administration of NK-4. On the other hand, plasma might serve as a sink that enhances clearance of Aβ from the brain based on a peripheral sink hypothesis [35], [36], because Aβ clearance mainly occurred in plasma in Tg2576 mice [34]. It might be also possible that direct NK-4 binding to Aβ creates a sink that enhances clearance of Aβ deposition from brain. In either case, further study is required to address these issues.

It seems unlikely that the impaired performance of Tg2576 mice in learning and memory tests is due to changes in motivation or sensorimotor function, since the motivation for each behavioral test is different and different skills are required for a good performance in each test. There were no differences in locomotor activity, total time spent exploring objects in the novel object test between wild type and Tg2576 mice. Recognition memory in the novel object test and associative learning in the passive avoidance test are dependent on the hippocampus and/or perirhinal cortex [37]–[39]. In this context, progressive impairment of the hippocampus and/or perirhinal cortex-dependent memory started as early as 6 months of age and continued to 12 months of age in Tg2576 mice in our experiment. Contrary to this, histological investigation of Aβ deposits in Tg2576 mice showed no substantial differences in quantity of CR-positive plaques among the saline-treated group and NK-4-treated groups at 12 month of age (Fig. 8a). On the other hand, Aβ-immunoreactive tangles were abundant in the cortex of saline-treated Tg2576 mice and they were clearly decreased by high dose NK-4 treatment in good agreement with the results of Aβ ELISAs (Fig. 7, Fig. 8b, c), and it might also reflect the cognitive function (Fig.3, 4). The difference in plaque stainability between CR and anti-Aβ would be due to the sensitivity to immature tangles, and therefore, CR staining might not reflect the total amount of Aβ deposition in brain.

The Tg2576 mouse is the most thoroughly characterized AD mouse model and is considered to reflect the human amyloid pathology most closely among mouse models [11]. Although many studies using mice on a B6/SJL background (hereafter; Tg2576/B6), we used here the newly developed 129S6/SvEvTac strain (hereafter; Tg2576/129) established by Taconic, because this strain does not carry a retinal degeneration mutation [24]. The 129 strains are reported not appropriate for water maze tasks according to previous studies [40]–[43], however, the wild type mice of 129S6/SvEvTac strain performed well in the acquisition learning of water maze test [44]. Regarding the timing of cognitive decline, our results showed that the Tg2576/129 did not exhibit cognitive decline at the age of 3 months (data not shown), but became apparent at 6 months, which continued to 12 months (Fig. 3,4,5). Reportedly, Tg2576/B6 showed no detectable cognitive impairment at 3–4 months, but became obvious at 5–6 months of age [14], [45]–[47]. Thus, genetic backgrounds appear not to be affected in the timing of cognitive decline.

The water maze protocol we employed in this study is a variant of Morris water maze navigation task. Although the original protocol was designed for evaluating hippocampus-dependent spatial reference learning and memory [48], our modified protocol is not considered as the one for spatial cognition since we used extra intra-maze landmarks and did not run a probe trial. We placed proximal landmarks on the inner rim of the pool in addition to each wall of the room (distal landmarks) aiming to enhance positional recognition of mice, however, it is suggested that the processing of information related to distal landmarks (room cues) or proximal landmarks (intra-maze cues) are mediated by different neural systems [49], [50]. The hippocampus circuitry contributes to a spatial strategy, which involves flexible use of spatial/distal cues, whereas a cue strategic processing of proximal landmarks is dependent upon striatal circuitry [50]. Thus, what we evaluated using the extra proximal landmark condition would not be spatial reference learning, but non-spatial or mixture of both. On another note, the lack of probe trial made it difficult to confirm the memory retention. As a result of these modified experimental design, we could not determine whether the learning strategy of the mice was spatial or not. One thing for certain is, however, that NK-4 modulated the learning ability of Tg2576/129 mice in the water maze task (Fig.4). The learning ability of Tg2576/129 mice was impaired with age, and a long-term NK-4 treatment significantly improved it. Results of two other behavioral tests (Fig.3, Fig.5) support this notion.

In relation to the learning ability of the mice on 129 background, it has been known that all 129 mouse substrains have disrupted-in-schizophrenia 1 (*DISC1*) deletion mutation [51]. Because *DISC1* is reported to play a critical role in development and migration of hippocampal neurons [52], cognitive deficits observed in 129 strains may linked to lack of DISC1 protein. In this regard, preferable effect of NK-4 on cognitive improvements of Tg2756/129 mice may be attributable to restoring the dysfunctional *DISC1*-mediated pathway. To address this question, we examined the effect of NK-4 on Aβ-dependent amnesic mice model [25]. As a result, NK-4 treatment dose-dependently and significantly attenuated memory dysfunction induced by direct injection of Aβ_25–35_ peptide into the lateral ventricle of ICR mice (Fig. 9). Therefore, the modification of *DISC1*-mediated pathway by NK-4 seems not mainly involved in this case. Since NK-4 activated PI3K-Akt pathway independently of the TrkA receptor *in vitro* [20], activated Akt (p-Akt) may be an important mediator of the beneficial effects on learning and memory. The p-Akt is required for PI3K-mediated synaptic plasticity and memory consolidation by promoting neuronal cell survival and protein synthesis via phosphorylation of CREB, a downstream regulator involved in hippocampus-dependent long-term and spatial memory formation [53]. Therefore, induction of Akt phosphorylation might play critical roles in NK-4-mediated memory improvement in Tg2576/129 mice.

Oxidative mechanisms are also thought to be involved in the cell loss and other neuropathology associated with AD [54], [55]. In AD pathogenesis, reactive oxygen species (ROS) impair mitochondrial redox activity and increases further ROS generation [29], [56], [57]. Aβ induces production of ROS and leads to apoptotic neuronal cell death that can be inhibited by antioxidants [57]–[59]. Pathologic and biochemical studies suggest that ROS induced by fibrillar Aβ have neurotoxic effects [60], [61]. As mentioned before, NK-4 is a potent scavenger of ROS [21] and an inhibitor of Aβ aggregation (Fig. 2). Those activities might contribute to attenuation of Aβ toxicity in AD.

As to the safety profile, NK-4 was well tolerated in a mouse acute oral toxicity study at up to 2 g/kg [21], based on mortality, clinical observations, body weight, hematology, blood chemistry, organ weights, and histological examinations of a full tissue list. Additionally, NK-4 was not mutagenic in the standard Ames test due to bacterial-specific metabolism (data not shown). Furthermore, there were no specific adverse events in mice those received intraperitoneal injections of NK-4 at the dose of up to 500 µg/kg/day, 5 days a week, for 9 months (this study). These observations suggest that NK-4 is a safe compound that will not cause serious adverse reactions. Following an intraperitoneal administration at 500 µg/kg, NK-4 penetrated the brain and nM levels were detected using high-performance liquid chromatography [20]. Thus, the nM range used in the *in vitro* investigations in this study corresponds to the calculated brain concentration in Tg2576/129 mice receiving NK-4.

In summary, the results of this study show that chronic treatment with NK-4 from an early stage of cognitive dysfunction significantly ameliorated learning impairments in Tg2576/129 AβPP mouse. The NK-4-induced behavioral improvement might be attributable to decreased Aβ accumulation in brain, and the inhibitory effects on Aβ aggregation, neurotrophin-like property, as well as radical-scavenging effect of NK-4. Its multiple functions might work in concert to attenuate AD pathology.
